# The Zika Virus Individual Participant Data Consortium: A Global Initiative to Estimate the Effects of Exposure to Zika Virus during Pregnancy on Adverse Fetal, Infant, and Child Health Outcomes

**DOI:** 10.3390/tropicalmed5040152

**Published:** 2020-09-30

**Authors:** Zika Virus Individual Participant Data Consortium

**Keywords:** Zika virus, individual participant data meta-analysis, data sharing, emerging pathogen, prognostic model, prediction model, congenital Zika syndrome, microcephaly

## Abstract

This commentary describes the creation of the Zika Virus Individual Participant Data Consortium, a global collaboration to address outstanding questions in Zika virus (ZIKV) epidemiology through conducting an individual participant data meta-analysis (IPD-MA). The aims of the IPD-MA are to (1) estimate the absolute and relative risks of miscarriage, fetal loss, and short- and long-term sequelae of fetal exposure; (2) identify and quantify the relative importance of different sources of heterogeneity (e.g., immune profiles, concurrent flavivirus infection) for the risk of adverse fetal, infant, and child outcomes among infants exposed to ZIKV in utero; and (3) develop and validate a prognostic model for the early identification of high-risk pregnancies and inform communication between health care providers and their patients and public health interventions (e.g., vector control strategies, antenatal care, and family planning programs). By leveraging data from a diversity of populations across the world, the IPD-MA will provide a more precise estimate of the risk of adverse ZIKV-related outcomes within clinically relevant subgroups and a quantitative assessment of the generalizability of these estimates across populations and settings. The ZIKV IPD Consortium effort is indicative of the growing recognition that data sharing is a central component of global health security and outbreak response.

While the world has turned to face COVID-19, recent epidemics are re-emerging [1,2]. Even as vector control is sidelined to confront the current crisis [3], close to 3 billion Zika virus (ZIKV) naïve people live in *Aedes-aegypti* endemic areas in Asia and Africa [4]. Despite the advances in ZIKV research, researchers are only now beginning to characterize the longer-term sequelae of fetal ZIKV exposure [5,6]. Identification of factors that affect the risk of fetal infection and adverse pregnancy, birth, and pediatric developmental outcomes is central to quantifying the burden of disease related to ongoing and future ZIKV transmission and critical for the development of diagnostic assays, vaccines, appropriate early intervention strategies, and targeted preventive measures.

In February 2017, a group of researchers formed the ZIKV Individual Participant Data (IPD) Consortium to conduct an individual participant data meta-analysis (IPD-MA) of ZIKV-related longitudinal studies of pregnant women and their children. The ZIKV IPD Consortium initiative arose from a meeting of international organizations and Ministries of Health in June 2016 to coordinate and harmonize ZIKV-related research efforts [7]. IPD-MA is the synthesis and analysis of participant-level data across related studies and provides a number of statistical and clinical advantages over aggregate data meta-analysis [8]. The research objectives of the ZIKV IPD Consortium IPD-MA are to (1) estimate the absolute and relative risks of miscarriage, fetal loss, and short- and long-term sequelae of fetal Zika exposure for women that experience symptomatic and asymptomatic ZIKV infection during pregnancy; (2) identify and quantify the relative importance of different sources of heterogeneity in the risk of adverse fetal, infant, and child outcomes among infants exposed to ZIKV in utero; and (3) develop and validate a prognostic prediction model [9] to identify high risk pregnancies and inform communication between health care providers and their patients and to optimize mobilization of resources (e.g., vector control strategies, antenatal care, and family planning programs). The IPD-MA will include data from longitudinal studies or country-level active surveillance systems that measure ZIKV exposure and infection during pregnancy and subsequent fetal, infant, and/or child outcomes. The full protocol for the IPD-MA is available from *BMJ Open* (https://bmjopen.bmj.com/content/9/6/e026092).

As of August 2020, 52 cohort studies and country-level active surveillance system sites from 28 countries and territories have agreed to contribute de-identified data to the ZIKV IPD Consortium IPD-MA. The use of participant-level data from diverse geographies and populations will allow the team to better identify the clinical, virological, environmental, and individual-level factors that predict severe ZIKV-related fetal effects or adverse neurocognitive or neurodevelopmental outcomes in the child, several of which can only be measured after two years of age [10]. For example, the diversity of populations will help researchers understand differences in the sequelae of fetal exposure between populations with a high prevalence of recent prior exposure to other arboviruses and those where ZIKV occurs alone, while accounting for yellow fever immunization. Figure 1 shows the distribution of ZIKV cases at the global level, extracted from the PAHO PLISA Health Information Platform for the Americas [11]. Each circle represents a longitudinal study or country-level active surveillance system with circle size corresponding to the number of pregnant women enrolled and the line indicating the time period during which studies recruited pregnant women. As indicated in the graph, the number of new ZIKV cases has decreased dramatically since 2018, and several of the larger studies began towards the end of the epidemic in the Americas. Utilizing available data will be especially important since few studies have been able to secure funding for the follow-up needed to understand the spectrum and incidence of longer-term consequences of fetal exposure to ZIKV.

The global response to ZIKV has been characterized by unprecedented levels of collaboration between countries, research organizations, universities, and surveillance systems [12]. The ZIKV IPD Consortium effort is indicative of the growing recognition that data sharing is a central component of global health security and outbreak response. Ideally, cross-study and cross-system data synthesis and analysis would take place in real time during an epidemic. To make that possible, the governance and systems for data and sample sharing need to be in place before the next major outbreak. Statistical methods for valid synthesis and analysis of large combined datasets in an IPD-MA need to be adapted to inform the public health response to emerging pathogens [13], especially when there is no reference standard that can be used to evaluate the relative accuracy of different diagnostic assays [14,15,16].

Women living in and travelling to countries with ongoing or recent ZIKV transmission face a tremendous burden from the fear and uncertainty around the risks associated with ZIKV infections during pregnancy, guidance from public health authorities to avoid or delay pregnancy, and the lack of economic and social support for the challenges of raising infants with congenital Zika syndrome (CZS) and other ZIKV-related disabilities and developmental delays [17,18]. Research study participants have the added burden of intensive follow-up that may require frequent blood draws and long commutes. While there is currently no vaccine or prophylaxis to prevent or mitigate the effects of ZIKV infection during pregnancy, leveraging data across studies to improve precision and developing the statistical methods needed to improve the accuracy and quantify the uncertainty of the information conveyed to families and health systems are ethical imperatives.

Despite the persistent threat of ZIKV infection to pregnant women and women of reproductive age worldwide, investment in critical areas of ZIKV research is waning. Leveraging existing investments through collaboration will accelerate the development of effective prevention, harm reduction, and control strategies. Best practices and lessons learned from the research response to ZIKV can be used to improve the coordination and speed of the global research response to emerging pathogens.

Global travel, trade, urbanization, and climate change have all facilitated the development of novel hosts [19] and extended the range of existing hosts [20], fundamentally altering the geographic range of vector-borne diseases. Innovations in research response are central to ensuring that global health security systems keep pace. The synthesis of participant-level data across studies with the ZIKV IPD Consortium IPD-MA is a first step. Employing prospective harmonization and real-time collaborative analyses that utilize participant-level data to inform epidemic response is the way forward.

## Figures and Tables

**Figure 1:**
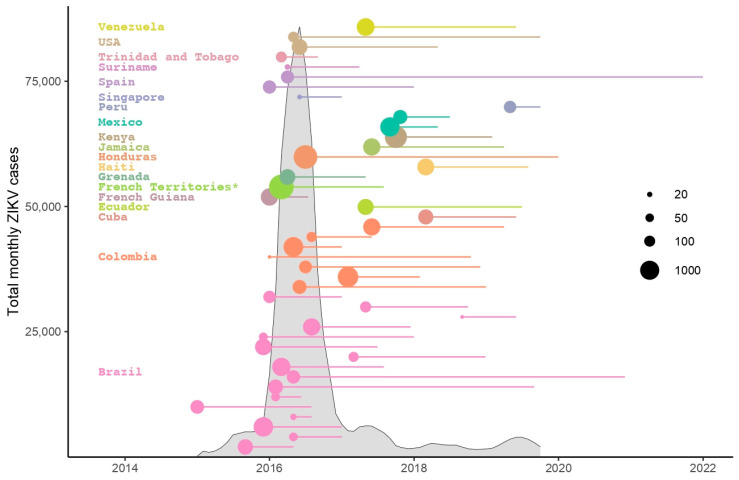
Distribution of monthly Zika virus (ZIKV) cases and Zika virus-related longitudinal studies of pregnant women and their infants and children. Vertical axis shows the total number of monthly reported ZIKV cases for countries/regions listed (6-month moving average of raw data extracted from PAHO PLISA Health Information Platform for the Americas, 2 November 2019). Each of the 42 colored circles represents a participating study with color corresponding to country/region and circle size corresponding to the number of pregnant women enrolled in the study. The horizontal colored lines indicate the time period during which a participating study recruited pregnant women. Ten studies missing information on number of pregnant women enrolled are excluded from the graph. * French Territories include Guadeloupe, Martinique, and French Guiana.

## Zika Virus Individual Participant Data Consortium members

Jackeline Alger, Ricardo Arraes de Alencar Ximenes, Vivian I. Avelino-Silva, Azucena Bardají, Carlos Hernan Becerra Mojica, Andrea Benedetti, Maria de Lourdes Benamor Teixeira, Sarah Bethencourt, Victor Hugo Borja Aburto, Fátima Brant, Patrícia Brasil, Elizabeth B. Brickley, Nathalie Broutet, Pierre Buekens, María Luisa Cafferata, Guilherme Calvet, Harlan Campbell, Mabel Carabali, Derrick Chan, Federico Costa, Orlando da Costa Ferreira, Conrado Milani Coutinho, Antonio Jose Cunha, Carlos Cure Cure, Johanna A.A. Damen, Valentijn M.T. de Jong, Thomas P. Debray, Roberta L. DeBiasi, Luis Alfonso Diaz-Martinez, Geraldo Duarte, Diana María Ferriol, Jucelia S. Ganz, Patrick Gérardin, Suzanne M. Gilboa, Maritza Gonzalez, Concepción Grajales Muñiz, Paul Gustafson, Luz Angela Gutierrez Sanchez, María G. Guzmán, Cristina Hofer, Natanael Holband, Irene Inwani, Thomas Jaenisch, Esaú João, Amadu Juliana, Edna Kara, Caron Kim, Albert I. Ko, Marion Koopmans, Angelle Desiree LaBeaud, Maura Lash, Ellen H. Lee, Yee-Sin Leo, Brooke Levis, Nicola Low, Calum N.L. Macpherson, Elena Marbán-Castro, Salim Mattar, Lauren Maxwell *, Philippe Mayaud, Adriana Melo, Clara Menéndez, Marcela Mercado Reyes, María Consuelo Miranda Montoya, Demócrito de Barros Miranda-Filho, Karel G.M. Moons, Ivonne Morales, Maria Elisabeth Moreira, Sarah B. Mulkey, José Esteban Muñoz Medina, Marisa Marcia Mussi-Pinhata, Muktha S. Natrajan, M. Kariuki Njenga, Trevor P. Noel, Mauricio Nogueira, Eric Osoro, Martha Lucia Ospina Martinez, Marc Paladini, Saulo Passos, Freddy Perez, Léo Pomar, Arnaldo Prata-Barbosa, Mitermayer Reis, Ludovic Reveiz, Carlota Rodó, Luiza Emylce Pelá Rosado, Kerstin D. Rosenberger, Nuria Sanchez Clemente, Janet L. Sayers, Antonio A. Silva, Isadora C. de Siqueira, Karen Sohan, Carmen Soria-Segarra, Antoni Soriano-Arandes, Patricia Sousa, João Paulo Souza, Anna Suy Franch, Adriana Tami, Mauro Teixeira, Soe Soe Thwin, Van T. Tong, Marília Dalva Turchi, Celina Maria Turchi Martelli, Diana Valencia, Maria D. Van Kerkhove, Thalia Velho Barreto de Araújo, Luis Angel Villar, Carmen Viñuela Benéitez, Yinghui Wei, Marc-Alain Widdowson and Annelies Wilder-Smith.

## Affiliations

Facultad de Ciencias Médicas Universidad Nacional Autónoma de Honduras, Tegucigalpa 11101, Honduras; (J.A.), Departamento de Medicina Tropical da Universidade Federal de Pernambuco, Recife 50670-901, Brazil; (R.A.d.A.X.), Medical School São Paulo, Departamento de Molestias Infecciosas e Parasitarias University of São Paulo, São Paulo 01000, Brazil; (V.I.A.S.), Maternal, Child, and Reproductive Health Initiative, Barcelona Institute for Global Health (ISGlobal), Hospital Clinic—University of Barcelona, Barcelona 08036, Spain; (A.B. (Azucena Bardají)); (E.M.C.); (C.M.), Departamento de Obstetricia y Ginecología, Universidad Industrial de Santander, Bucaramanga 680002, Colombia; (C.H.B.M.); (L.A.G.S.), McGill University Health Centre, McGill University, Montréal, QC H4A 3J1, Canada; (A.B. (Andrea Benedetti)), Department of Infectious Diseases, Hospital Federal dos Servidores do Estado, Rio de Janeiro 22440-901, Brazil; (M.d.L.B.T.); (O.d.C.F.); (E.J.), Facultad de Ciencias de la Salud, Universidad de Carabobo, Valencia 2005, Venezuela; (S.B.), Unidad de Atención Primaria a la Salud, Instituto Mexicano del Seguro Social, Ciudad de México 06600, México; (V.H.B.A.), Universidade Federal de Minas Gerais, Minas Gerais 31270-901, Brazil; (F.B.); (M.T.), Acute Febrile Illnesses Laboratory, Evandro Chagas National Institute of Infectious Diseases, Oswaldo Cruz Foundation, Rio de Janeiro 20231-050, Brazil; (P.B. (Patrícia Brasil)); (G.C.), Department of Infectious Disease Epidemiology, London School of Hygiene and Tropical Medicine, London WC1E 7HT, UK; (E.B.B.), NDP/UNFPA/UNICEF/WHO/World Bank Special Programme of Research, Development and Research Training in Human Reproduction, Department of Sexual and Reproductive Health and Research, World Health Organization, 1211 Geneva 27, Switzerland; (N.B.); (E.K.); (C.K.); (L.M.); (J.L.S.); (S.S.T.), School of Public Health and Tropical Medicine, Tulane University, New Orleans LA 70112, USA; (P.B. (Pierre Buekens)), Mother and Children Health Research Department, Institute for Clinical Effectiveness and Health Policy, Buenos Aires C1056ABH, Argentina; (M.L.C.), Department of Statistics, University of British Columbia, Vancouver BC V6T 1Z4, Canada; (H.C.); (P.G. (Patrick Gérardin)), Epidemiology, Biostatistics and Occupational Health, McGill University, Montreal QC H3A 1A2, Canada; (M.C.), Paediatric Neurology, Paediatric Medicine, KK Women’s and Children’s Hospital, 229899 Singapore; (D.C.), Institute of Collective Health, Federal University of Bahia, Salvador 40110-909, Brazil; (F.C.), Departmento de Ginecologia e Obstetrícia, Hospital das Clínicas da Faculdade de Medicina de Ribeirão Preto da Universidade de São Paulo, São Paulo 65470-000, Brazil; (C.M.C.), Departamento de Pediatria, Federal University of Rio de Janeiro, Rio de Janeiro 21941-901, Brazil; (A.J.C.), BIOMELAB SAS, Barranquilla 080001, Colombia; (C.C.C.), Julius Center for Health Sciences and Primary Care, UMC Utrecht, Utrecht University, Utrecht 3584 CG, The Netherlands; (J.A.A.D.); (V.M.T.d.J.); (K.G.M.M.), Department of Epidemiology, University Medical Center Utrecht, Utrecht 3584 CX, The Netherlands; (T.P.D.), Departments of Pediatrics and Microbiology, Immunology and Tropical Medicine, The George Washington University School of Medicine, Children’s National Research Institute, Washington, DC 20052, USA; (R.L.D.), Departamento de Pediatría, Universidad Industrial de Santander, Bucaramanga 680002, Colombia; (L.A.D.M.), Ribeirão Preto Medical School of University of São Paulo, São Paulo 14049-900, Brazil; (G.D.), Centro Provincial de Genética Médica, Cienfuegos 90100, Cuba; (D.M.F.), Children’s Hospital Juvencio Matos, São Luís 65065-545, Brazil; (J.S.G.), Centre for Clinical Investigation (CIC1410), Centre Hospitalier Universitaire de La Réunion, Saint Pierre 97448, Réunion, France; (P.G. (Paul Gustafson)), National Center on Birth Defects and Developmental Disabilities, Centers for Disease Control and Prevention, Atlanta, GA 30333, USA; (S.M.G.); (V.T.T.); (D.V.), Instituto Nacional de Salud—Colombia, Bogotá 111321, Colombia; (M.G.); (M.L.O.M.), División de Vigilancia Epidemiológica de Enfermedades Transmisibles, Instituto Mexicano del Seguro Social, Ciudad de México 06600, Mexico; (C.G.M.), Centro de Investigación, Diagnostico, y Referencia, Centro Colaborador OPS-OMS Para el Estudio del Dengue y su Control, Instituto de Medicina Tropical, Pedro Kourí (IPK), Havana, Cuba; (M.G.G.), Instituto de Puericultura e Pediatria Martagão Gesteira, Universidade Federal do Rio de Janeiro, Rio de Janeiro 21941-901, Brazil; (C.H.), Academic Pediatric Center Suriname, Academic Hospital Paramaribo, Paramaribo, Suriname; (N.H.); (A.J.), Department of Paediatrics and Child Health, Kenyatta National Teaching and Referral Hospital, Nairobi 00202, Kenya; (I.I.), Heidelberg Institute for Global Health, Heidelberg University Hospital, Heidelberg 69120, Germany; (T.J.), Department of Epidemiology of Microbial Diseases, Yale School of Public Health, New Haven, CT 06510, USA; (A.I.K.); (M.R.), Department of Virology, Erasmus MC, Rotterdam 3015 CN, The Netherlands; (M.K.), Pediatric—Infectious Diseases, Stanford Hospital, Palo Alto, CA 94304, USA; (A.D.L.B.), Bureau of Communicable Disease, NYC Department of Health and Mental Hygiene, New York, NY 11101, USA; (M.L.); (E.H.L.); (M.P.), Executive Director’s Office, National Centre for Infectious Diseases, 308442 Singapore; (Y.S.L.), Centre for Prognosis Research, School of Primary, Community and Social Care, Keele University, Keele ST5 5BG, UK; (B.L.), Institute of Social and Preventive Medicine, University of Bern, Bern 3012, Switzerland; (N.L.), Windward Islands Research and Education Foundation, St. George’s University, True Blue Point, Grenada; (C.N.L.M.); (T.P.N.), Institutos de Investigaciones Biologicas del Tropico, Universidad de Córdoba, Córdoba 14014, Colombia; (S.M.), Department of Clinical Research, London School of Hygiene and Tropical Medicine, London WC1E 7HT, UK; (P.M.), Instituto de Pesquisa Professor Joaquim Amorim Neto, Campina Grande, Paraiba 58402-040, Brazil; (A.M.), Research, Science, and Technology Division, Instituto Nacional de Salud—Colombia, Bogotá 111321, Colombia; (M.M.R.), Facultad de Salud Carrera, Universidad Industrial de Santander, Bucaramanga 680002, Colombia; (M.C.M.M.); (L.A.V.), Faculdade de Ciencias Medicas da, Universidade de Pernambuco, Recife 50670-901, Brazil; (D.d.B.M-F.), Department of Infectious Diseases, Heidelberg University Hospital, Heidelberg 69120, Germany; (I.M.); (K.D.R.), Department of Neonatology, Fundação Oswaldo Cruz (Fiocruz), Rio de Janeiro 20231-050, Brazil; (M.E.M.), Departments of Pediatrics and Neurology, The George Washington University School of Medicine, Fetal Medicine Institute, Children’s National Research Institute, Washington, DC 20052, USA; (S.B.M.), División de Laboratorios de Vigilancia e Investigación Epidemiológica, Instituto Mexicano del Seguro Social, Ciudad de México 06600, México; (J.E.M.M.), Departamento de Pediatria, Faculdade de Medicina de Ribeirão Preto da Universidade de São Paulo, São Paulo 14049-900, Brazil; (M.M.M.P.), Department of Medicine, Emory University, Atlanta, Georgia 30322, USA; (M.S.N.), Washington State University Global Health Program, Washington State University, Pullman, WA 99164 USA; (M.K.N.), Department of Dermatologic Diseases, Faculdade de Medicina de São José do Rio Preto, São José do Rio Preto 15090-000, Brazil; (M.N.), Paul G. Allen School for Global Animal Health, Washington State University, Nairobi, Kenya; (E.O.), Department of Pediatrics, Faculty of Medicine of Jundiaí, São Paulo 13202-550, Brazil; (S.P.), Communicable Diseases and Environmental Determinants of Health Department, Pan American Health Organization, Washington, DC 20037, USA; (F.P.), Department of Obstetrics and Gynecology, Centre Hospitalier de l’Ouest Guyanais 97320, Saint-Laurent du Maroni, French Guiana; (L.P.), Materno-fetal and Obstetrics Research Unit, Department Woman-Mother-Child, Lausanne University Hospital, Lausanne 1011, Switzerland, Department of Pediatrics, D’Or Institute for Research & Education, Rio de Janeiro 22281-100, Brazil; (A.P.B.), Gonçalo Moniz Institute, Oswaldo Cruz Foundation, Ministry of Health, Salvador 40296-710, Brazil; (M.R.); (A.I.K.), Evidence and Intelligence for Action in Health, Pan American Health Organization, Washington, DC 20037, USA; (L.R.), Maternal-Fetal Medicine Unit, Department of Obstetrics, Hospital Universitari Vall d’Hebron, Barcelona 08035, Spain; (C.R.), Hospital Materno Infantil de Goiânia, Health State Secretary, Goiás 74125-090, Brazil; (L.E.P.R.), Department of Infectious Disease Epidemiology, London School of Hygiene and Tropical Medicine, London WC1E 7HT, UK; (N.S.C.), Department of Public Health, Federal University of Maranhão, São Luís 65080-805, Brazil; (A.A.S.), Instituto Gonçalo Moniz, Fundação Oswaldo Cruz (Fiocruz), Salvador 40296-710, Brasil; (I.C.d.S.), Obstetrics and Gynaecology, Diagnostic (Mother & Baby) Ltd., Lange Park 120110, Trinidad and Tobago; (K.S.), SOSECALI C. Ltd.a. Universidad Católica Santiago de Guayaquil, Guayaquil 090-615, Ecuador; (C.S.S.), Department of Pediatrics, University Hospital Vall d’Hebron, Barcelona 08035, Spain; (A.S.A.), Reference Center for Neurodevelopment, Assistance, and Rehabilitation of Children, State Department of Health of Maranhão, Sao Luís 65076-820, Brazil; (P.S.), Department of Social Medicine, University of São Paulo, São Paulo 01000-000, Brazil; (J.P.S.), Obstetrics Department, Vall d’Hebron Hospital, Barcelona 08035, Spain; (A.S.F.), Department of Medical Microbiology, University Medical Center Groningen, University of Groningen, Groningen 9712 CP, The Netherlands; (A.T.), Departamento de Parasitología Médica, Facultad de Ciencias de la Salud, Universidad de Carabobo, Valencia 2005, Venezuela, Institute of Tropical Pathology and Public Health, Federal University of Goias, Goiânia 74690-900, Brazil; (M.D.T.), The Research Centre Aggeu Magalhães, Fundação Oswaldo Cruz (Fiocruz), Recife 50670-420, Brazil; (C.M.T.M.), Department of Infectious Hazards Management, World Health Organization, 1211 Geneva 27, Switzerland; (M.D.V.K.), Department of Social Medicine, Federal University of Pernambuco, Recife 50670-420, Brazil; (T.V.B.d.A.), Obstetrics & Gynecology, Hospital General Universitario Gregorio Marañón, Madrid 28007, Spain; (C.V.B.), Centre for Mathematical Sciences, University of Plymouth, Plymouth PL4 8AA, UK; (Y.W.), Division of Global Health Protection, US Centers for Disease Control and Prevention, Nairobi, Kenya; (M.A.W.), Institute of Tropical Medicine, 2000 Antwerp, Belgium, Department of Disease Control, London School of Hygiene and Tropical Medicine, London WC1E 7HT, UK; (A.W.S.).
